# Pseudoprogression in lung cancer: a case report

**DOI:** 10.37349/etat.2020.00022

**Published:** 2020-10-30

**Authors:** Giulia Meoni, Nicola Libertà Decarli, Maurizio Benucci, Claudio Raspanti, Angela Stefania Ribecco

**Affiliations:** 1 Medical Oncology Unit, Oncology Department, San Giovanni di Dio Hospital, Azienda USL Toscana Centro, 50143 Florence, Italy; 2 Pathology Unit, Oncology Department, San Giovanni di Dio Hospital, Azienda USL Toscana Centro, 50143 Florence, Italy; 3 Reumatology Unit, Department of Internal Medicine, San Giovanni di Dio Hospital, Azienda USL Toscana Centro, 50143 Florence, Italy; 4 Interventional Radiology Unit, Diagnostic Radiology Department, San Giovanni di Dio Hospital, Azienda USL Toscana Centro, 50143 Florence, Italy; Università Politecnica Marche, Italy

**Keywords:** Non-small cell lung cancer, immunotherapy, pseudoprogression, performance status, kirsten rat sarcoma

## Abstract

Immunotherapy dramatically changed the management of several malignancies including non-small cell lung cancer (NSCLC). Since immune checkpoint inhibitors have a different mechanism of action from cytotoxic agents or small molecules against NSCLC, also tumor response may present with atypical features. Pseudoprogression (PP) is a distinct response pattern defined by a transient enlargement of the tumor burden, sustained by inflammatory cells and usually not associated with worsening of performance status (PS). Here the authors describe the case of a lung adenocarcinoma patient treated with pembrolizumab, who developed an early symptomatic PP with a dramatic global worsening of PS. Subsequently an improvement in general condition and a brilliant tumor response were observed. Tumor re-biopsy was collected after the treatment in order to support the identification of PP and to describe microenvironment modifications induce by immunotherapy.

## Introduction

Immunotherapy profoundly modified the therapeutic algorithm of several malignancies including non-small cell lung cancer (NSCLC) [1]. Monoclonal antibodies against programmed death 1 (PD1) and programmed death 1 ligand (PD-L1) are called immune checkpoint inhibitors (ICIs). ICIs are able to avoid negative regulatory signals on immune system and to restore an effective immune response against tumor cells [2, 3]. Since ICIs have a different mechanism of action from cytotoxic agents or antitumor small molecules, also tumor response may present with atypical features [4, 5]. Due to this reason, response evaluation criteria in solid tumor (RECIST) version 1.1-which are regularly used to assess the efficacy of anticancer agents-are not fully applicable to immunotherapy [6]. This led to the formulation of specific response evaluation criteria such as immune-related response criteria (irRC) proposed in 2009 [7], immune-related RECIST (irRECIST) and immune RECIST (iRECIST), developed respectively in 2013 and 2017 [8, 9]. The major difference from RECIST version 1.1 is that, per immune criteria, progressive disease (PD) needs to be confirmed by a repeat consecutive assessment at least 4 weeks after the previous computed tomography (CT) scan. Moreover, as per immune-modified RECIST (imRECIST) proposed in 2018 [10], patients are allowed to continue immunotherapy even in the case of PD documented at a subsequent assessment, if they do not have any deterioration in performance status (PS) or signs or symptoms of unequivocal PD. This new concept of PD introduced by immune criteria reflects a new pattern of treatment response, which is pseudoprogression (PP) [11]. PP occurs in up to 6% of NSCLC patients treated with ICIs and it is defined by an initial transient enlargement of the tumor burden and/or the development of new lesions, sustained by the massive recruitment of immune cells surrounding the tumor [12]. PP usually occurs without a worsening of patient PS, a short-term radiologic follow-up performed after 4–8 weeks documenting the therapeutic response allows clear differentiation with true PD. Even more atypical and challenging is the case in which patients with PP experience clinical deterioration, due to the vigorous activation of the immune system and the mass effect of enlarged sites of disease [13]. This clinical scenario can be called symptomatic PP and it is hardly to be distinguished in the short term from another uncommon pattern of immune response which is hyperprogressive disease (HPD). HPD incidence ranges from 4% to 29% and it can be conceived as a dramatic increase in tumor burden (at least 50%) with a rise in progression pace greater than twofold and a rapid clinical worsening [14]. Here we describe the case of a lung adenocarcinoma kirsten rat sarcoma (*KRAS*)-mutated patient treated with pembrolizumab, an anti-PD1 agent, who developed an early symptomatic PP with a dramatic global worsening of PS. Few weeks later, patient PS improved and an impressive radiologic tumor response was documented at radiological follow-up. In order to support the identification of PP and to describe microenvironment modifications induced by immunotherapy, tumor re-biopsy was collected after treatment.

## Case Report

**Figure 1.**
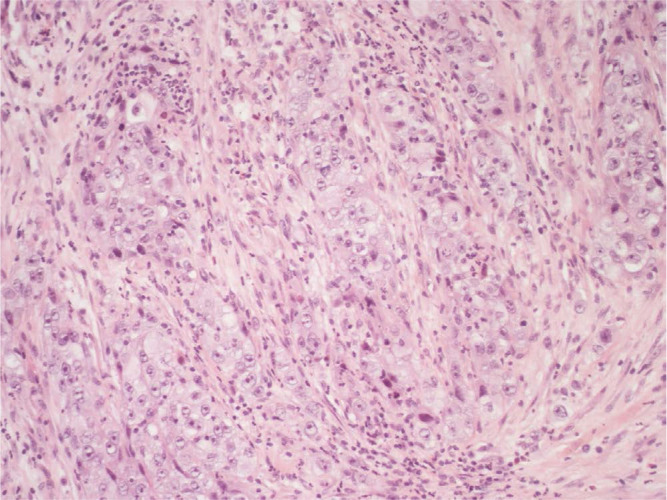
Right supraclavicular lymphnode biopsy (first sending): fibrous tissue infiltrated by poorly differentiated adenocarcinoma (hematoxylin-eosin, 20X)

**Figure 2.**
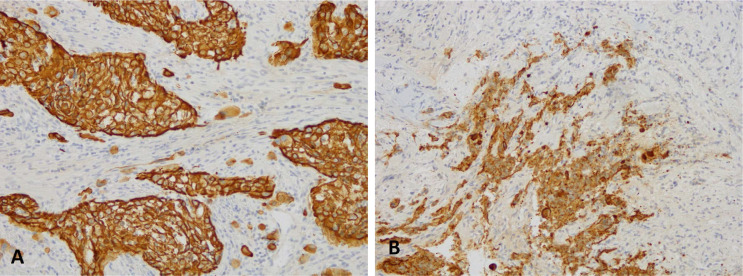
Immunohistochemical analysis for PDL-1 (clone sp263 Ventana) highlights a high positivity in the neoplastic cells: the picture on the left (A) shows the analysis performed on the right supraclavicular lymphnode, first sending; the picture on the right (B) shows the analysis performed on the right supraclavicular lymph node, second sending (10X)

**Figure 3.**
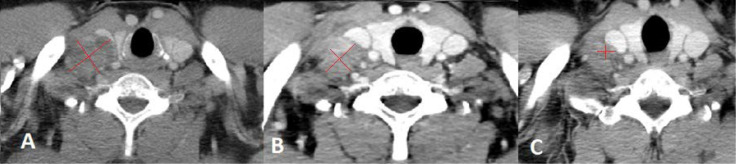
Chest CT scan performed at baseline (A), after the first cycle of Pembrolizumab (B) and after the fourth cycle of therapy (C). Image A shows the enlarged supraclavicular lymphnode 2.7 cm x 3.8 cm, compressing the right internal jugular vein. Image B shows the right supraclavicular lymphadenopathy moderately reduced in size 2.5 cm x 3.2 cm, with less evident signs of compression on the internal jugular vein. Image C shows a further dimensional reduction of the right supraclavicular lymphnode 1.04 cm x 1.15 cm, which is more than halved with respect to baseline

**Figure 4.**
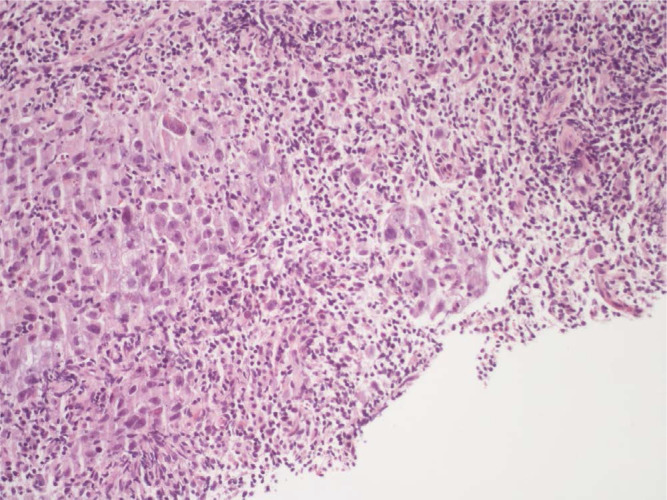
Right supraclavicular lymphnode biopsy (second sending): fibrous tissue infiltrated by poorly differentiated adenocarcinoma (on the left of the picture). It should be noted the presence of an increase in the lymphocyte component in the intra-peritumoral inflammatory cells infiltrate (hematoxylin-eosin, 20X)

**Figure 5.**
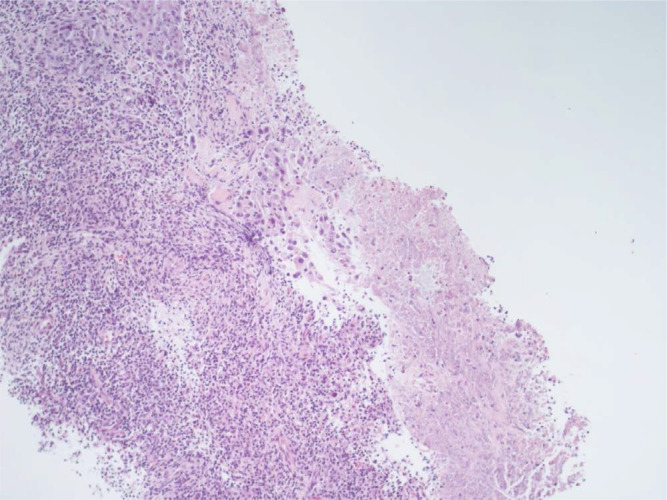
Right supraclavicular lymphnode biopsy (second sending). An area of necrosis is noted on the right of the picture, while on the there is an infiltration of poorly differentiated adenocarcinoma with associated a discrete component of the accompanying lymphocyte infiltrate (hematoxylin-eosin, 10X)

A 51-year-old man presented to our clinic due to the appearance of a palpable mass in the right supraclavicular region. The patient had an Eastern cooperative oncology group (ECOG) PS of 0. He denied cough, fever, weight loss, pain and he was a former smoker. He was affected by obliterating arteriopathy of the lower limbs and he had undergone femoral stent placement few months before. A right supraclavicular lymph node biopsy was performed and histological examination revealed metastasis from transcriptional thyroid factor 1 (TTF1) positive lung adenocarcinoma (Figure 1). Next generation sequencing (NGS) tumor DNA examination was conducted using QIamp DNA FFPE Tissue Kit for DNA estraction (Qiagen) and QIact Actionable Insight Tumor Panel on FFPE for HotSpot gene analysis (Qiagen). The presence of *KRAS* p. G12C mutation was documented, no additional alterations were identified by NGS. Immunohistochemistry revealed PD-L1 expression in 80% of tumor cells (Figure 2). CT scan of the thorax and abdomen demonstrated the presence of lymphadenopaties in the right supraclavicular region 3.8 cm x 2.7 cm infiltrating the jugular vein (Figure 3), at right hilar and right paratracheal stations, respectively 1.0 cm x 1.8 cm and 3.8 cm x 2.7 cm, with trachea resulting compressed by the lymph node mass. The primary lung tumor was not clearly detectable. A fluorodeoxyglucose positron emission tomography (FDG PET) CT documented hypermetabolic lymphadenopaties corresponding to the known sites of disease [standard uptake value (SUV)_max_ of 6] and no suspected visceral nor bone metastases. Brain magnetic resonance imaging (MRI) was negative. Therefore the disease stage was cTxN3M0 (TNM 8thedition). On January 2020 first line treatment with Pembrolizumab 200 mg i.v. q3w was started. Few days after the first course of therapy, the patient showed a dramatic size increase of the right supraclavicular lymphadenopathy which resulted at least doubled in size, painful and with erytematous and edematous skin. The patient showed an ECOG PS of 3 and complained severe hyporexia, asthenia, dysphagia, dysphonia and recurrent syncopal episodes. During the next 3 weeks symptoms and general condition progressively improved, the patient recovered to an ECOG PS of 0 and the right supraclavicular lymph node mass significantly shrinked. A thorax-abdomen CT scan and a supraclavicular lymph node re-biopsy were performed immediately before the second administration of pembrolizumab. CT scan revealed dimensional reduction of the thoracic lymphadenopaties, configuring partial response (PR) as per RECIST version 1.1 (Figure 3). Supraclavicular lymph node re-biopsy revealed a localization of poorly differentiated adenocarcinoma of compatible lung origin, with inflammatory infiltration mainly consisting of T-lymphocytes and extensive areas of coagulation necrosis (Figures 4 and 5). Based on clinical, radiological and histological findings, diagnosis of symptomatic PP was made and the patient continued the treatment with pembrolizumab. The CT scan performed after the 4th cycle documented a further dimensional reduction of the thoracic lymphadenopaties (Figure 3) and the FDG PET CT revealed a single residual hypermetabolic site of disease in the right supraclavicular region with a lower tumor metabolic activity (SUV_max_ of 3.5). A stereotactic body radiation therapy (SBRT) on the FDG-avid lymhadenopathy was planned. Nevertheless, as the patient underwent CT simulation, the supraclavicular mass was no more detectable due to a further reduction in burden of disease and SBRT was omitted. Actually the patient is continuing pembrolizumab therapy and as of July 2020 received 7 cycles overall, with a good tolerability.

## Discussion

**Figure 6.**
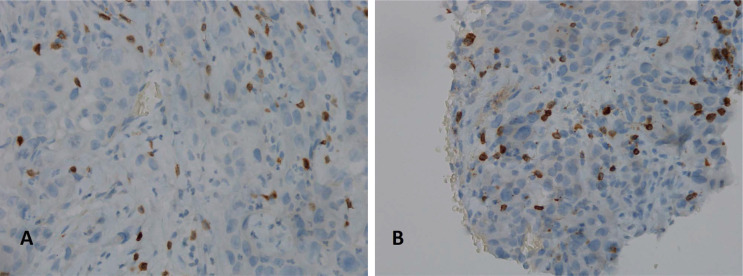
Immunohistochemical analysis for CD3 (clone 2GV6 Ventana): the picture on the left (A) shows the analysis performed on the right supraclavicular lymph node, first sending; the picture on the right (B) shows the analysis performed on the right supraclavicular lymph node, second sending (40X). T-lymphocytes (CD3+) are quite rare and scattered among the neoplastic cells

**Figure 7.**
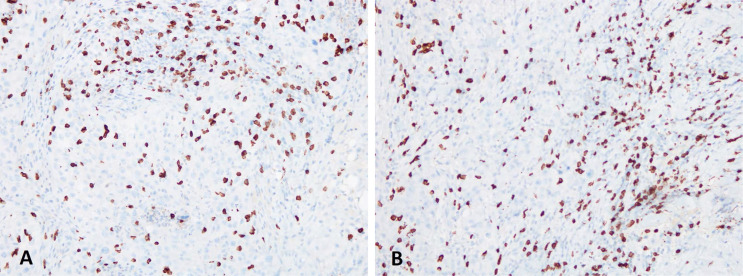
Immunohistochemical analysis for CD8 (clone SP57 Ventana): the picture on the left (A) shows the analysis performed on the right supraclavicular lymph node; the picture on the right (B) shows the analysis performed on the right supraclavicular lymph node (20X). T-lymphocytes (CD8+) show a zonal distribution

Here we report the case of a *KRAS*-mutated lung adenocarcinoma with an early symptomatic PP. PP was characterized by a rapid and massive dimensional increase of all sites of disease and a vigorous activation of the immune system. After few weeks from the first pembrolizumab infusion, symptoms and PS improved and CT scan with a lymph node re-biopsy documented PP of disease. It is crucial to distinguish PP from a true PD and even more challenging is to recognize a symptomatic PP from a HPD, due to the considerable similarities of the respective clinical presentations. Nevertheless these differential diagnoses are of utmost importance, in order to avoid both a premature discontinuation of an effective treatment and the delay of starting a new therapy. Usually the PS deterioration reflects PD or HPD, whilst clinical benefit is a marker of treatment efficacy. Nevertheless, case reports described patients with PP experience clinical deterioration mainly due to two reasons: the mass effect of enlarged sites of disease which are diffusely infiltrated by immune cells and the extensive systemic inflammatory response [11, 13]. The mass effect may be eventually responsible for the onset of dyspnea, cough, dysphagia, dysphonia and pain, depending on the enlarged sites of disease. The hyperactivation of the immune response produces a sort of cytokines storm which may cause fever, hyporexia, asthenia and sweats. Based on these evidences, PS should be considered for clinical decisions but should not be used to definitively judge the efficacy of immunotherapy. Rather PS, biopsy of enlarged lesions, radiographic follow-up and FDG PET represent all complementary references for clinical decision making. Biopsy of enlarged lesions represents certainly the gold standard for PP diagnosis. In fact, the major mechanism of PP is the infiltration of immune cells which can be revealed by the re-biopsy. Pathologic findings may include immune infiltrate with lymphocytes positive for CD3/CD4/CD8 and a decreased CD4/CD8 ratio. Macrophagocytes, tumor necrosis, hemorrhage, edema and no viable tumor cells may be also observed [15, 16]. In our case the first sample of the patient is a biopsy of a right supraclavicular lymph node and is represented by a fragment of fibrous tissue with localization of poorly differentiated adenocarcinoma. On immunohistochemical analysis, the tumor cells are positive for keratin 7 and TTF1, supporting a pulmonary origin of the neoplasm (Figure 1). The PDL-1 expression (clone sp263 Ventana) is present in the 80% of neoplastic cells (Figure 2). The inflammatory component is mainly represented by neutrophilic granulocytes, while T-lymphocyte cells (CD3+; CD8+) are rare and macrophagocytes are absent (Figures 6 and 7). There is no evidence of necrotic areas. Two months later, a re-biopsy of the same lymph node was performed (Figures 4 and 5). In this case extensive areas of coagulation necrosis were observed. On the periphery of these areas, nests of neoplastic cells were recognized, compatible with poorly differentiated adenocarcinoma. The inflammatory component mainly consists of T-lymphocytes (CD3+; CD8+) with a conserved CD4/CD8 ratio, rare macrophagocytes were observed (Figures 6 and 7). About immunohystochemical analysis with PDL-1, still 80% of the neoplastic cells resulted positive (Figure 2). Beside tumor re-biopsy, circulating tumor DNA (ctDNA) seems to be a promising non-invasive tool to monitor tumor response after ICIs [17–21]. Few published studies support the correlation between decreased or low-level ctDNA and PP *versus* higher ctDNA level in true PD [20, 21]. This observation could allow to differentiate PP from true PD through a non-invasive liquid biopsy. Interestingly, some evidence exists among the correlation between ctDNA in *KRAS*-mutated lung adenocarcinoma and PP [21], which is the case of our patient. Guibert et al. [21], monitored the levels of *KRAS*-mutated ctDNA in two patients who had experienced PP and compared the variations with those from of a patient who had true PD. Authors found that ctDNA showed rapid and dramatic decrease in patients with PP, whilst it was strongly increased in the patient with true PD. This lead to the conclusion that ctDNA may be an additional non-invasive and promising tool to discriminate PP from true PD for tumors that harbor an oncogenic addiction. Our patient developed such a vigorous immune response that a higher sensibility to the immunotherapic drug can be hypothesized. Some authors suggest that these “hypersensitive” patients may be overtreated when receiving the currently indicated dosage every three weeks [13]. Nevertheless, actually neither the time and the intensity of immune response nor the onset of clinical activity of ICIs are predictable yet. In our case, PP earlier developed either at the supraclavicular level or in the mediastinum, considering the symptoms complained by the patient. But in some cases PP involves only some sites of disease and not the entire tumor burden, it may be delayed or even occur repeatedly during the course of the disease in a totally unpredictable manner [22–24]. Our patient showed early treatment efficacy with a dimensional reduction of tumor burden and also a brilliant metabolic response. This may suggest a better prognosis when comparing this pattern of response, sustained by such a massive inflammatory infiltrate, with respect to stable disease (SD) or PR. Some retrospective evidence exists that patients experiencing PP may carry a better prognosis in terms of 12 months overall survival (OS) with respect to patients developing SD or PR as per RECIST version 1.1 [12]. Actually there is not a consensus upon this issue and further analyses are required. In conclusion, symptomatic PP represents an atypical pattern of response which can be observed after immunotherapy. PP differential diagnosis with true PD and HPD is crucial. PS, tumor re-biopsy, close radiographic follow-up and potentially ctDNA analysis represent complementary references for clinical decision making in such a challenging clinical scenario.

## Abbreviations

CT: computed tomography
ctDNA: circulating tumor DNA
ECOG: Eastern cooperative oncology group
FDG PET: fluorodeoxyglucose positron emission tomography
HPD: hyperprogressive disease
ICIs: immune checkpoint inhibitors
*KRAS*: kirsten rat sarcoma
NGS: next generation sequencing
NSCLC: non-small cell lung cancer
PD: progressive disease
PD1: programmed death 1
PD-L1: programmed death 1 ligand
PP: pseudoprogression
PR: partial response
PS: performance status
RECIST: response evaluation criteria in solid tumor
SBRT: stereotactic body radiation therapy
SD: stable disease
SUV: standard uptake value
TTF1: transcriptional thyroid factor 1

## Declarations

### Copyright

© The Author(s) 2020.
